# Antimicrobial Resistance Patterns in *Clostridioides difficile* Strains Isolated from Neonates in Germany

**DOI:** 10.3390/antibiotics9080481

**Published:** 2020-08-04

**Authors:** Friederike K. M. T. Tilkorn, Hagen Frickmann, Isabel S. Simon, Julian Schwanbeck, Sebastian Horn, Ortrud Zimmermann, Uwe Groß, Wolfgang Bohne, Andreas E. Zautner

**Affiliations:** 1 Institute for Medical Microbiology, University Medical Center Göttingen, 37075 Göttingen, Germany; f.tilkorn@stud.uni-goettingen.de (F.K.M.T.T.); isabelsimone.simon@stud.uni-goettingen.de (I.S.S.); julian.schwanbeck@med.uni-goettingen.de (J.S.); ortrudzimmermann3@gmail.com (O.Z.); ugross@gwdg.de (U.G.); wbohne@gwdg.de (W.B.); 2 Department of Microbiology and Hospital Hygiene, Bundeswehr Hospital Hamburg, 20359 Hamburg, Germany; frickmann@bnitm.de; 3 Institute for Medical Microbiology, Virology and Hygiene, University Medicine Rostock, 18057 Rostock, Germany; 4 Department of Pediatrics, University Medical Center Göttingen, 37075 Göttingen, Germany; sebastian.horn@med.uni-goettingen.de; 5 Department of Pediatrics, SRH Central Hospital Suhl, 98527 Suhl, Germany

**Keywords:** *Clostridioides difficile*, children, Germany, antibiotic resistance, selection

## Abstract

Young children are frequently colonized with *Clostridioides* (*C.*) *difficile*. Depending on their resistance patterns, antibiotic treatment can facilitate gastrointestinal spreading in colonized individuals, potentially leading to transmission to others. *C. difficile* was isolated from stool samples from infants born in two hospitals in Göttingen and Darmstadt, Germany. All isolates were subjected to phenotypic antimicrobial resistance testing, PCR-based screening for toxin genes and mass spectrometry-based exclusion of ribotypes 027 and 176. Within an initial cohort of 324 neonates with a longitudinal survey of *C. difficile*, 137 strains were isolated from 48 individuals. Antimicrobial resistance was recorded against metronidazole in one (0.7%), erythromycin in 16 (11.7%) and moxifloxacin in 2 (1.5%) of the strains, whereas no resistance was observed against vancomycin (0.0%) or rifampicin (0.0%). Newly observed resistance against erythromycin in children with detection of previously completely sensitive isolates was reported for *C. difficile* isolates from 2 out of 48 children. In 20 children (42%), non-toxigenic strains were detected, and from 27 children (56%), toxigenic strains were isolated, while both toxigenic and non-toxigenic strains were recorded for 1 child (2%). Ribotypes 027 or 176 were not observed. In conclusion, the German *C. difficile* strains isolated from the children showed mild to moderate resistance with predominance of macrolide resistance, a substance class which is frequently applied in children. The observed switches to the dominance of macrolide-resistant isolates suggests likely selection of resistant *C. difficile* strains already in children.

## 1. Introduction

While toxigenic *Clostridioides (C.) difficile* strains can cause severe colitis usually in elderly patients, especially if associated with antimicrobial chemotherapy affecting the gut microbiome [1,2,3], asymptomatic colonization of the human gut with this microbe is frequent in neonates and infants [4,5,6,7,8,9,10]. Age-dependent impairment of the immune system is discussed as a major factor responsible for this reduced vulnerability of and pathogenicity in children [11,12]. 

In adults, in contrast, pathogenicity of *C. difficile* is mediated by toxins A and B. The genes *tcdA* and *tcdB* of those toxins are located on the pathogenicity locus *PaLoc*. Transcription of these genes is regulated by a positive regulator gene (*tcdR*), an inhibitory regulator gene (*tcdC*), and a gene responsible for extracellular release of the toxins (*tcdE*) [13,14]. The toxins A and B act as glycosyltransferases. First, they bind to surface receptors of the intestinal epithelial cells and reach the interior of the cells via receptor-mediated endocytosis. Within the cells, they inactivate Rho GTPases by glycosylation, which are involved in the metabolism of the actin elements of the cytoskeleton. As a result of this, deformation of the cells and damage to tight junctions lead to the loss of cell–cell contacts. In addition, the inactivation of the Rho GTPases facilitates increased formation of pro-apoptotic factors and thus cell death [13,14]. The extent to which early childhood colonization with toxigenic strains as well as the associated formation of antibodies against toxins A and B lead to protection against symptomatic infections in later life is a subject of ongoing research [15].

However, since children can harbor *C. difficile* in their gut, and especially if the usual hygiene precautions are neglected, they can spread the pathogen via the fecal–oral route, and thus they are a potential source of transmission [8]. Thereby, antibiotic treatment of the children can facilitate the selection of resistant strains in the gut [16,17,18].

To quantify the phenomenon of antibiotic resistance in colonizing *C. difficile* strains in neonates in German hospitals associated with the presence or absence of toxins and the highly pathogenic ribotypes 027 and 176 [19,20], a study was conducted at the University Medical Center of Göttingen, Germany, and at the Children’s Clinic Princess Margaret Darmstadt, Germany.

In detail, the study aimed at recording resistance patterns and potential changes in the course of a longitudinal assessment, potential associations with the highly pathogenic ribotypes 027 and 176 and the abundance of pathogenicity factors in strains from German neonates. By doing so, a piece of the epidemiological puzzle of *C. difficile* abundance and resistance in asymptomatic neonates should be provided.

## 2. Materials and Methods

### 2.1. Study Population and Patient Samples

The study protocol was approved by the institutional ethics board of the University Medical Center Göttingen (Application number 22/11/16). During a 22-month period, stool samples were collected from 324 neonates born at the University Medical Center Göttingen, Germany, and the Children’s Hospital Princess Margaret, Darmstadt, Germany. While several thousand neonates had been born in the study centers during the study interval, only the parents of those 324 individuals agreed to participate in the study. The study was conducted as an open cohort assessment with longitudinal recruitment of neonates from birth to the end of the study 22 months after its start. Beginning with the first stool after birth (meconium), samples of each neonate were sent by the parents to the microbiological laboratory at the University Medical Center Göttingen in a monthly interval until the 6th month of life. In some instances, this procedure could be continued until the 12th month of life. Individual missing samples were no exclusion criteria. The samples were acquired by research sampling without clinical indication of diarrhea.

### 2.2. Isolation of C. difficile

For all stool samples obtained, bacterial culture of *C. difficile* was performed at 37 °C for about 48 h in an anaerobic atmosphere using a COY anaerobic gas chamber (COY Laboratory Products, Grass Lake, MI, USA), with an atmosphere consisting of 85% N_2_, 10% H_2_ and 5% CO_2_, and selective chromogenic chromID^®^ *C. difficile* agar (bioMérieux, Nürtingen, Germany). Subsequently, the isolated bacterial strains were transferred to Columbia agar plates enriched with 5% sheep blood (bioMérieux) for further assessment. Species identification by matrix-assisted laser desorption/ionization time-of-flight mass spectrometry (MALDI-TOF-MS) was performed using an Autoflex III smart beam mass spectrometer (Bruker Daltonics, Bremen, Germany).

### 2.3. Antimicrobial Resistance Testing

Antibiotic resistance testing was performed with *C. difficile* suspensions in 0.9% NaCl at an optical density OD_600_ of 0.1 on Mueller Hinton agar enriched with 5% sheep blood at an anaerobic atmosphere with E-tests “MIC Test Strip” (Liofilchem^®^, Roseto degli Abruzzi, Italy) with metronidazole, vancomycin, erythromycin, moxifloxacin and rifampicin as described by the manufacturer. Applied resistance definitions were as follows: MIC (minimum inhibitory concentration) >2.0 µg/mL for vancomycin according to EUCAST (European Committee on Antimicrobial Susceptibility Testing) version 10.0, MIC > 2 µg/mL for metronidazole according to EUCAST version 10.0, MIC > 8 µg/mL for moxifloxacin according to CLSI (Clinical and Laboratory Standards Institute, M100S, 26th ed. 2016), MIC > 4 µg/mL for erythromycin according to DIN (“Deutsches Institut für Normierung”) version DIN 58940-1:2002-10 and MIC > 16 µg/mL for rifampicin according to [21].

### 2.4. PCR Targeting Toxin Genes

After nucleic acid extractions from culture materials using a Magna Pure LC 2.0 (Roche Diagnostics, Mannheim, Germany) automate, PCR for *C. difficile* associated genes of toxin A and B was performed using the GenoTypeCDiff Version 1.0 kit (Hain Lifescience, Nehren, Germany) and additionally confirmed applying the Real Star*^®^ Clostridium difficile* PCR Kit 1.0 (altona Diagnostics, Hamburg, Germany) as described by the manufacturers.

### 2.5. Proteotyping for Detection of Ribotype 027 and 176

The highly pathogenic ribotypes 027 and 176 were excluded by proteotyping using the Autoflex III smart beam mass spectrometer (Bruker) as described recently [22].

## 3. Results and Discussion

In total, 137 *C. difficile* isolates were recovered from a longitudinal follow-up of 48 out of 324 (14.8%) neonates.

### 3.1. Recorded Antimicrobial Resistance

**Figure 1:**
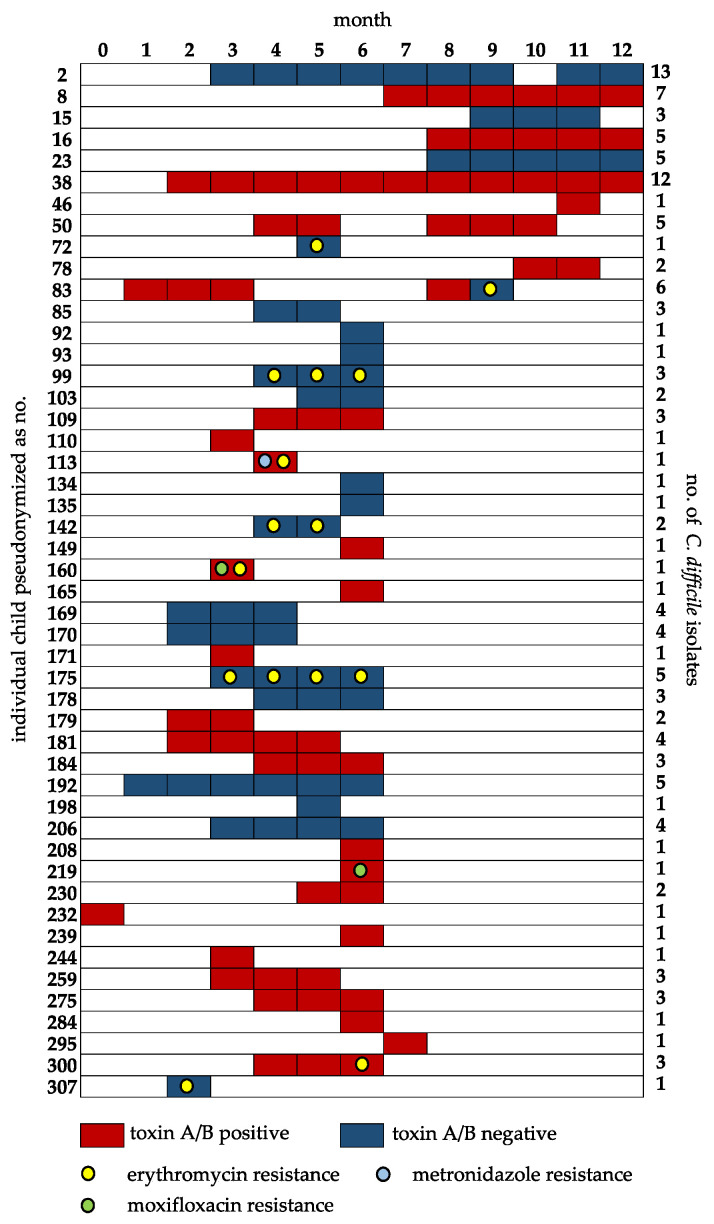
Overview of the presence of *C. difficile* strains, toxin A/B genes and antibiotic resistance over time. The left axis indicates the pseudonymized child number. The number on the right axis denotes the total number of isolated strains from that child. Strain presence is indicated by colored boxes. Toxin A/B-positive strains are colored red; negative strains are colored blue. Antibiotic resistances are indicated as colored dots. Note: Only in child 83, a switch from previously toxin A/B-positive isolates to toxin A/B-negative isolates, which showed erythromycin resistance, occurred from month 8 to month 9. In child 300, there was a switch to erythromycin-resistant and toxin A/B-positive isolates at month 6. All other children showed a constancy in colonization regarding the examined parameters.

According to the resistance definitions applied, no resistant strain was detected for vancomycin and rifampicin. Metronidazole resistance was recorded in 1/137 isolates (0.7%, recorded MIC > 256 µg/mL), moxifloxacin resistance in 2/137 isolates (1.5%, recorded MICs > 32 µg/mL each) and erythromycin resistance in 16/137 isolates (11.7%, recorded MICs 2 × 16–24 µg/mL, 1 × 64 µg/mL, 13 × >256 µg/mL). A concurrent resistance against erythromycin and moxifloxacin, as well as a concurrent resistance against erythromycin and metronidazole were observed in one strain each. In two neonates, erythromycin resistance in the colonizing *C. difficile* strains was observed at the age of 6 months (child 300) and 9 months (child 83), respectively, while susceptible *C. difficile* strains had been isolated in the course of the previous assessments at the ages of 4 and 5 months as well as 1, 2, 3 and 8 months, respectively (Figure 1). No treatment with erythromycin was documented for any of the infants during the study period.

### 3.2. Recorded Distribution of Toxin Genes

Positive PCR results for toxin A/B were recorded in 73 out of 137 (53.3%) isolates from 27 out of 48 (56.3%) neonates colonized with *C. difficile*. Solitary detections of the genes for toxin B as well as detections of the gene for the binary toxin did not occur. Concordance of both applied PCR assays was 100%. Just in one neonate (child 83), a toxin gene-negative *C. difficile* isolate was detected at the age of 9 months, while toxin gene-positive strains had been isolated at the ages of 0, 1, 2, 3 and 8 months, respectively (Figure 1). No other changes in strain characteristics were detected.

### 3.3. Proteotyping-Based Assessment for the Ribotypes 027 and 176

Among the assessed 137 strains, the ribotypes 027 and 176 were not detected.

### 3.4. Discussion

The assessment indicated colonization with *C. difficile* in 14.8% of screened neonates at two German hospitals. This result is generally in line with previous reports indicating high colonization rates with *C. difficile* in neonates [4,5,6,7,8,9,10]. The prevalence we found is comparatively in the low to moderate range. More than half of the isolates harbored toxin genes, suggesting likely etiological relevance in the case of nosocomial transmission to susceptible hosts. In line with this, other authors also stressed the importance of asymptomatically colonized neonates carrying *C. difficile* as potential sources for hidden dispersion of the pathogen [8,10].

Resistance was comparably mild and focused on the macrolide erythromycin, while moxifloxacin and metronidazole resistances were rare, and vancomycin as well as rifampicin resistances were absent. Applicability of macrolides even in neonates makes selection processes under antibiotic pressure hypothetically possible. Although no antimicrobial drugs were administered in the study cohort, transmission of unsusceptible strains could occur through contact with other children or adults treated with erythromycin. Macrolides may have been administered to the mother in the prenatal phase and thus resistant *C. difficile* strains may have been selected and transferred from mother to child. This might have been of particular relevance in the two neonates with first detections of erythromycin-resistant strains after several months. As suggested by international literature, resistance of *C. difficile* patient isolates shows a geographically dispersed pattern. While resistance against therapeutic drugs of choice in the case of *C. difficile*-associated colitis like metronidazole and vancomycin show low resistance rates of 0.1% and 2.3%, respectively, in Europe [21], 15.6% metronidazole-resistant strains were observed in a Chinese assessment [23] and 17.9% vancomycin-resistant isolates in an US American study [24].

As suggested by the proteotyping results, highly pathogenic ribotypes like 027 and 176 were absent in the assessed population of German neonates. Again, the literature indicates timely and geographical heterogenicity regarding the colonization of neonates and infants with ribotype 027. While several studies are in line with the here-described absence of the ribotype in this age group [8,25], an American study reported up to 20% ribotype 027 among strains isolated from children [26]. In some studies, the validity of ribotype 027 as a cause of particularly severe courses of *C. difficile*-associated colitis is controversially discussed [27,28]. Complete PCR ribotyping of all strains from this assessment was impossible due to financial constraints, which is an admitted limitation of this study.

The study is further limited by the lack of availability of data on the family environment (especially regarding the antibiotic treatment of family members), making interpretations on the likely ways of transmission and reasons for the observed minor gain of resistance hypothetical. To reduce potential bias due to specificity limitations of the applied diagnostic tools [29,30], the reliability of the PCR for the toxin genes was confirmed by a second PCR assay. Another limitation is the lacking availability of data on strains from adult patients from the study sites.

Summarized, the assessment confirms low to moderate colonization rates in German neonates with toxigenic *C. difficile* strains, underlining their potential role in the spreading of *C. difficile* if standard hygiene procedures are neglected. Among the detected resistance types, macrolide resistances are particularly noteworthy. The occurrence of macrolide resistance in this age cohort is probably due to the use of macrolides in children, pregnant, or lactating women and the associated selection processes in the intestinal flora.

## 4. Conclusions

In the present study, 137 *C. difficile* isolates from stool samples of 48 children were examined for the presence of antibiotic resistance, toxin genes and the ribotypes 027 and 176. The most frequently detected antibiotic resistance was erythromycin resistance with 11.7% (16/137), which can be explained by the fact that this antibiotic is used to treat bacterial infections in both infants and pregnant women. The very low resistance rates to the antibiotics metronidazole (0.7%) and vancomycin (0%), which are commonly used in the treatment of *C. difficile* infections, are consistent with the results of comparable studies in both children and adults. Toxigenic strains were detected in 27 children (56%), which is also consistent with results from previous studies. Only in two cases, a change in the resistance pattern or toxin expression could be detected. Accordingly, it can be assumed that the *C. difficile* strains persist as part of the child’s microbiome. Ribotypes 027 and 176 were not detected in the entire test cohort.
